# USPIO enhanced 4D flow imaging of the mouse cardiovascular system at 7T with an ultrashort echo time sequence

**DOI:** 10.1186/1532-429X-17-S1-O70

**Published:** 2015-02-03

**Authors:** Aurélien J Trotier, Charles Castets, William Lefrançois, Jean-Michel Franconi, Eric Thiaudière, Sylvain Miraux

**Affiliations:** 1 Centre de Résonance Magnétique des Systèmes Biologiques, Bordeaux, France

## Background

Time resolved phase contrast imaging is hardly used in small animal studies due to a lack of signal-to-noise ratio. Some studies were performed in 2D and one publication (Janiczek et al, MRM 2011) reported flow measurement in mouse aortic arch in 3D due to the use of a thin excitation slab and high Time-Of-Flight (TOF) effect. However, this strategy is not applicable on the whole cardiopulmonary system. To overcome this problem we proposed to combined Ultra-short Echo Time sequence with an injection of Ultra Small Particles of Iron Oxide (USPIO) to generate a stable, positive and high blood signal.

The method was first validated on phantom and then applied on the whole cardiopulmonary system of normal mice.

## Methods

The flow sequence was based on a 3D-cine non spatially selective UTE sequence (TR/TE =6/0.031 ms ). Flow encoding gradient (duration : 0.540 ms, Hadamard encoding method) were inserted between the RF pulse and imaging gradient, resulting in an echo time of 0.571 ms. The sequence was compared to a standard Phase Contrast cartesian FLASH sequence (TR/TE = 2.3/6 ms).

Flow measurements were performed on a rectilinear tube at 7T with an imposed average velocity of 25,8 cm/s. The tube was next fulfilled with various solution of MnCl2 (0/1/2/4/6/8 mM) to evaluate the robustness of the method as a function of r2 and r2*.

4D flow imaging of the mouse cardiovascular system was performed with a 100 µL injection of USPIO at a dose of 100 µmol Fe/kg with a ECG triggered UTE sequence (isotropic spatial resolution = 156 µm, images per cardiac cycle : 9, number of projections : 18144, acquisition time : 45 min).

## Results

On phantom, the measured velocity was in accordance with the imposed flow, using the UTE sequence, at each MnCl2 concentration, whereas an error superior to 5 cm/s was observed with the FLASH sequence when MnCl2 concentration was higher than 1mM.

**Figure 1:**
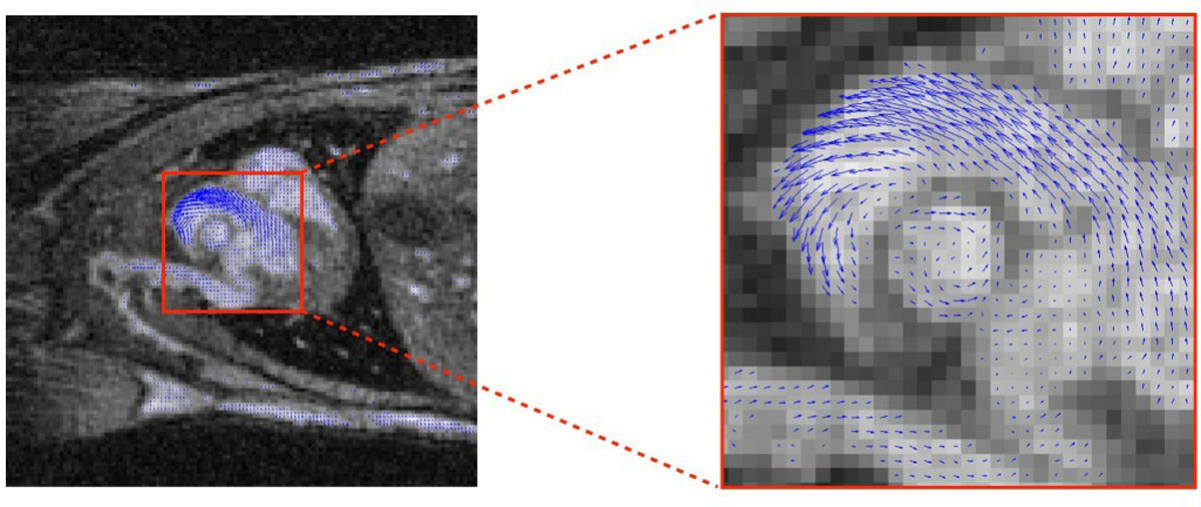
Extracted coronal slice with corresponding flow velocities vectors on heart.

On the mouse heart, UTE images were obtained without flow dephasing artefacts on the whole heart and in major vessels (aortic cross, carotids, pulmonary arteries, descending aorta). A blood velocity vector map in the aortic cross is presented in figure 1 and can be obtained in all vessels of the mouse.

## Conclusions

We have demonstrated that combining the injection of iron nanoparticles with 3D Time-Resolved Phase Contrast UTE sequences generated a strong positive contrast between blood and surrounding tissues. These properties were exploited to quantify blood flow velocity of the cardiovascular system in small animals at high magnetic fields with a high spatial (< (200 µm)3) and temporal resolution (16 ms). This approach might be useful to assess flow velocities and flow velocity variations in cardio-vascular disease models.

## Funding

This work was supported by a public grant, Translational Research and Advanced Imaging Laboratory, which is part of the French National Research Agency's Investments for the Future Program ("NewFISP"; ANR- 10-LABX-57).

